# Preeclampsia serum upregulates CD40/CD40L expression and induces apoptosis in human umbilical cord endothelial cells

**DOI:** 10.1186/1477-7827-10-28

**Published:** 2012-04-18

**Authors:** Chun-feng Wu, Fu-dan Huang, Ren-fang Sui, Jing-xia Sun

**Affiliations:** 1 Department of Gynecology and Obstetrics, the First Affiliated Hospital, Harbin Medical University, Harbin, 150001, P. R. China; 2 Harbin Medical University, Harbin, 150001, P. R. China

**Keywords:** Preeclampsia, Endothelial dysfunction, CD40/CD40L, Human umbilical cord endothelial cells

## Abstract

**Background:**

The endothelial cell dysfunction observed in preeclampsia (PE) may be induced by CD40/CD40L signaling. This study investigated the role of CD40/CD40L in the pathogenesis of PE by comparing the effect of maternal serum obtained from healthy pregnant women and PE patients on HUVEC cell growth, apoptosis and CD40/CD40L expression.

**Methods:**

Maternal serum was obtained from 20 patients with PE (PE group) as well as 20 healthy pregnant women (control group). The human umbilical endothelial cell line, CRL1730, was cultured in the presence of maternal serum for 24, 48, and 72 h after which cell growth and apoptosis were assessed by MTT and flow cytometry analysis, respectively. CD40/CD40L expression was determined using flow cytometry and RT-PCR analyses.

**Results:**

As compared to CRL1730 cells treated with control sera, those treated with PE sera had altered morphology, decreased cell growth, increased apoptosis and greater CD40/CD40L protein and mRNA expression. Stimulation of CD40/CD40L protein and mRNA expression by PE sera was greatest at 24 h.

**Conclusions:**

PE sera may induce endothelial cell damage possibly through increased CD40/CD40L expression in early-onset PE. Further studies are necessary to determine the factor(s) in PE sera responsible for the observed changes in endothelial cell viability.

## Background

Early-onset preeclampsia (PE) occurs within 34 weeks of pregnancy [1], and is a major cause of maternal and perinatal morbidity and mortality [2,3]. Although ensuring maternal safety, early pregnancy termination, the only treatment for PE, often results in serious neonatal developmental complications and sequelae. Therefore, prevention and early detection of PE is crucial.

Although the etiology of PE remains unknown, endothelial cell dysfunction plays an important role in its pathogenesis [4] and may be mediated by CD40/CD40L signal transduction [5]. In porcine coronary arteries, sCD40L significantly decreased endothelium-dependent vasorelaxation and endothelial nitric oxide synthase (eNOS) mRNA expression and increased O_2_^-^[6]. Furthermore, increased soluble CD40L and upregulated CD40/CD40L expression were detected in the whole-blood platelets and monocytes of women with PE [7,8].

CD40, a transmembrane glycoprotein, and its ligand, CD40L, a member of the TNF superfamily of molecules, are widely distributed in human endothelial cells, smooth muscle cells, lymphocytes, and activated platelets [9]. CD40/CD40L plays an important role in antigen presentation and autoimmune disease [10], and its signaling induces the expression of adhesion molecules and metalloproteinases [11]. In addition, CD40-CD40L interactions transduce immune and inflammatory signals, promoting the generation of Th1 and Th2 cytokines and stimulating blood cells to produce IL-1β precursors and IL-β-converting enzymes [12]. Increased IL-1 activity prolongs the inflammatory responses, resulting in endothelial cell damage [13]. Furthermore, Lin *et al.*[5] confirmed the relationship between CD40/CD40L expression and oxidative endothelial cell damage in human umbilical vascular endothelial cells (HUVECs).

This study sought to explore the role of CD40/CD40L in the pathogenesis of early-onset PE by determining the effects of maternal serum obtained from patients with early-onset PE on CD40/CD40L signaling in the human umbilical endothelial cell line, CRL1730. Specifically, the effects of maternal serum isolated from PE patients and healthy pregnant women on CRL1730 viability, apoptosis, and CD40/CD40L expression were determined.

## Methods

### Criteria for selecting pregnant women and the collection of blood specimens

From October 2008 to May 2010, 20 patients with PE and 20 randomly selected healthy pregnant women were recruited at the First Clinical College of Harbin Medical University. Early-onset PE was diagnosed as described by Valensise *et al.*[14]. The following inclusion criteria for the PE group were used in the present study: pregnant at gestational age of 20 to 34 weeks, blood pressure ≥140/90 mmHg, urine protein ≥ 300 mg/24 h or immunoreactive urine protein with epigastric pain and headache. The participants were all primiparous with a single fetus, had no history of hypertension and renal disease before pregnancy, and were free of placenta previa, early placental abruption or other obstetric complications. Six hours after fasting, 10 mL venous blood was collected in all study participants. The serum was isolated by centrifugation at 3000 r/min for 5 min after which the supernatant was collected, aliquoted into 1.5 mL Eppendorf tubes, and stored at -80°C. This study was approved by the Institutional Ethics Committee of the First Affiliated Hospital, Harbin Medical University, and patient consent was obtained from each participant.

### The culture and treatment of CRL1730 human umbilical vein endothelial cells (HUVECs)

The human umbilical vein endothelial cell line, CRL1730, was purchased from ATCC (Manassas, VA). CRL1730 cells were cultured in F-12 K medium (ATCC) containing endothelial cell growth supplement (ECGS; Sciencell; Carlsbad, CA) and 10% fetal bovine serum (FBS; Gibco; Carlsbad, CA) in a 5% CO_2_, 37°C incubator. After reaching confluency, the cells were subsequently cultured at a 1:2 ratio and passaged every other day.

After reaching 80% confluency, the cells were synchronized by starvation in serum-free medium for 24 h. In the control group, the cells were cultured in medium containing 10% maternal serum obtained from healthy pregnant women for 24, 48 and 72 h. In the PE group, the cells were cultured in the medium containing 10% maternal serum obtained from early-onset PE patients for 24, 48 and 72 h.

### MTT assay

The cells were digested to form a single-cell suspension in medium containing 10% FBS before they were seeded onto 96-well plates at a density of 3000 cells/200 μL/well. After 24 h, the cells were cultured in serum-free medium for 24 h after which non-adherent cells were removed by washing with PBS. Cells from every sample were plated in 5 wells, and control wells were added into the medium with 10% of the corresponding maternal serum in addition to MTT (AMRESCO, Solon, OH) and dimethyl sulfoxide (DMSO; AMRESCO). After 24, 48, and 72 h, the medium was replaced with 200 μL new medium containing 10% FBS, and 20 μL MTT solution was added at a final concentration of 5 mg/mL. The cells were then incubated for 4 h before the supernatant was discarded after which 150 μL DMSO was added before shaking for 10 min until the crystals were fully dissolved. Absorbance was measured at 490 nm using a TECAN microplate reader (Männedorf, Switzerland).

### Annexin V staining to assess CRL1730 apoptosis

CRL1730 cells were digested with trypsin, and the cell suspension was adjusted with medium to 2.5 × 10^5^ cells/mL. Cells were isolated from a 1-mL cell suspension by centrifugation at 1000 r/min for 5 min after which they were washed with PBS. After centrifugation at 1000 r/min for 5 min, apoptosis was determined using the Fascalibur apoptosis kit (NeoBioscience, Cambridge, MA). Before measurement, 5 μL of Annexin V and 10 μL of propidium iodide (PI) were added for 10 min. Three negative control groups were included: one without dye, one with 5 μL Annexin V alone, and one with 10 μL PI alone.

### Flow cytometry to assess CD40/CD40L expression

After reaching 80% confluency in six-well plates, CRL1730 cells were digested with trypsin and collected by centrifugation at 1000 r/min for 5 min before washing twice with PBS to remove cell debris. The cells were resuspended in F-12 K culture medium containing 10% FBS, and the cell concentration was adjusted to 5 × 10^5^ cells/mL. Direct fluorescence detection was performed by incubating 100 μL of staining buffer (PBS with 2% FCS, 0.1% NaN_3_ and 5 μL of FITC-conjugated anti-CD40 or CD40L monoclonal antibody [BD Bioscience, USA]) for 30 min at 4°C before centrifugation at 1000 rpm for 5 min. The supernatant was discarded, and the cells were washed twice with cold PBS prior to centrifugation at 1000 rpm for 5 min. After fixation with 500 μL of 1% paraformaldehyde (PFA), CD40/CD40L expression was determined using flow cytometry (BD Biosciences) by counting 10,000 cells. Mean fluorescence intensity (MFI) was used to represent the level of CD40/CD40L expression in CRL1730 cells relative to the standard normal reference fluorescence intensity (the international unit of light intensity, Cd).

### Reverse transcription polymerase chain reaction (RT-PCR) analysis

Trizol (Molecular Research Center, Inc., Cincinnati, OH) one-step extraction was performed to isolate total RNA from CRL1730 cells. cDNA was obtained through reverse transcription using the Roche first-strand cDNA synthesis kit (Roche; San Francisco, CA). In addition to a dNTP mix (TaKaRa Biotechnology, Dalian, China) and Taq enzyme (TaKaRa), the following PCR primers were synthesized by Invitrogen (Shanghai): CD40, forward 5’-TTGGTGGTGGTGGTGTTG-3’ and reverse 5’-GCATCTGTGTATATGGCTTCC-3’ (124 bp); CD40L, forward 5’-CCTCTGCCACCTTCTCTG-3’ and reverse 5’-TCTTCTATCTTGTCCAACCTTC-3’ (213 bp); and GAPDH, forward 5’-ACGGATTTGGTCGTATTGGG-3’ and reverse 5’-TCCTGGAAGATGGTGATGGG-3’ (202 bp). Glyceraldehyde-3-phosphate dehydrogenase (GAPDH) mRNA expression was used as an internal reference. The PCR amplification reaction conditions were as follows: pre-denaturation at 94°C for 3 min, 30 amplification cycles at 94°C for 30 s, 63°C for 30 s (for CD40) or 60°C for 30 s (for CD40L), and 72°C for 30 s, and a final extension at 72°C for 7 min. Agarose gel electrophoresis (2%) was observed using an imaging system (AlphaInnotech, Johannesburg, South Africa). Density scanning of the electrophoretic bands was performed, and relative target mRNA expression was determined using the absorbance CD40/CD40L divided by that of GAPDH.

### Statistical methods

Independent sample t-tests, which compare the mean scores of two groups for a given variable, were used to compare the effect of baseline characteristics between the normal pregnant and PE groups. Data was presented as mean ± SD. Repeated ANOVA was used to examine the difference of each indices at 24, 48 and 72 h between the two groups. *P*-values < 0.05 were considered statistically significant. Statistic analyses were performed using SAS 9.2 statistics software (SAS Institute Inc., Cary, NC, USA).

## Results

### Participant baseline characteristics

Forty pregnant women, including 20 patients with PE (PE group) and 20 healthy pregnant women (control group), were enrolled in this study. As shown in Table 1, no significant differences in age, body weight and height, and BMI were found between the two groups. Furthermore, no difference in the gestation week during which the blood sample was obtained was observed (Table 1).

**Table 1:** Comparison of baseline characteristics between the normal and PE pregnant groups

	Control (N = 20)	PE (N = 20)	*P* value
Age (y)	27.50 ± 2.82	28.65 ± 3.20	0.235
Gestation (weeks)	31.60 ± 1.73	31.60 ± 1.73	0.229
Body weight (kg)	69.25 ± 7.20	70.10 ± 6.80	0.703
Body height (m)	1.61 ± 0.04	1.60 ± 0.03	0.383
BMI (kg/m^2^)	26.69 ± 2.90	27.36 ± 2.96	0.477

Continuous variables were presented as mean ± SD, and the groups were compared using Independent sample *t*-tests; PE, preeclampsia; BMI, body mass index

*P < 0.05

### Effects of normal pregnant and PE sera on CRL1730 cell morphology

**Figure 1:**
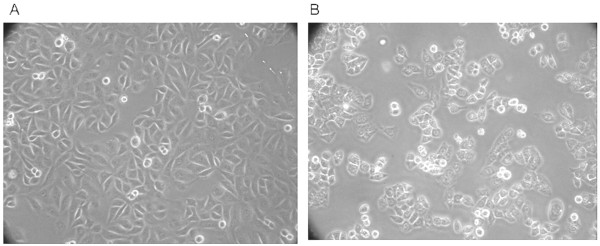
**Effects of normal pregnant and PE sera on CRL1730 cell morphology.** CRL1730 cell morphology upon stimulation with maternal sera obtained from A) normal pregnant and B) PE patients (300× magnification). A representative image from three independent experiments is shown.

As shown in Figure 1, the influence of patient sera on CRL1730 morphology was determined using inverted microscopy. CRL1730 cells cultured in the presence of control maternal sera were arranged in a mosaic-like monolayer; the cells were round and flat or polygonal with a cobblestone appearance (Figure 1A). CRL1730 cells cultured in maternal sera obtained from PE patients displayed altered morphology. Specifically, the cells were sparsely distributed with blurred boundaries between the nucleus and the cytoplasm; dark cytoplasmic granules were also observed (Figure 1B).

### Effects of normal pregnant and PE sera on CRL1730 growth and apoptosis

**Figure 2:**
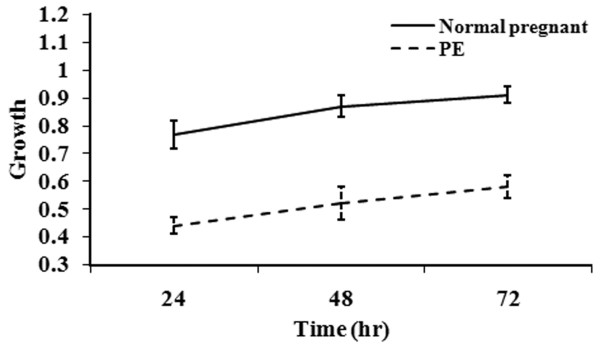
**CRL1730 cell growth after culture with sera obtained from the normal pregnant patients or those with PE.** Continuous variables were presented as mean ± SD and comparison of effect between two groups over time was performed by repeated measures of ANOVA and difference in growth between two groups over time was significant (P < 0.001). n = 20 for each group.

As shown in Figure 2, CRL1730 cell growth significantly increased over time in both groups (*P* <0.05). However, as compared with the cells treated with control maternal sera, CRL1730 cell growth in the PE group was significantly decreased (*P* <0.01). These data indicated growth inhibition by early-onset PE serum possibly due to cell damage.

**Figure 3:**
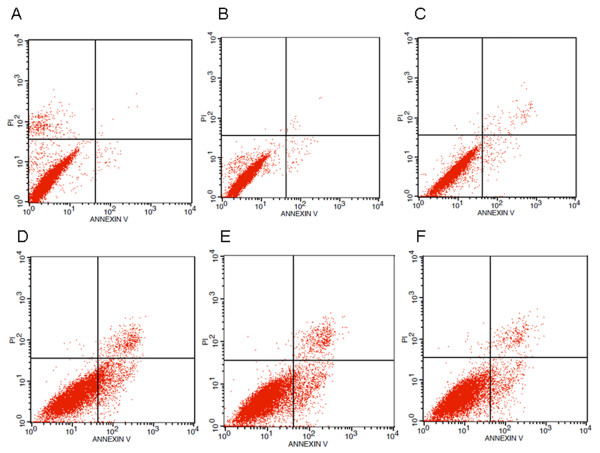
**Effects of normal and PE sera on CRL1730 cell apoptosis.** Apoptosis was determined in CRL1730 cells treated with serum from normal and PE pregnant women using Annexin V and PI double staining methods. A) 24-h normal pregnant group; B) 48-h normal pregnant group; C) 72-h normal pregnant group; D) 24-h PE group; E) 48-h PE group; and F) 72-h PE group. A representative image from three independent experiments is shown.

**Figure 4:**
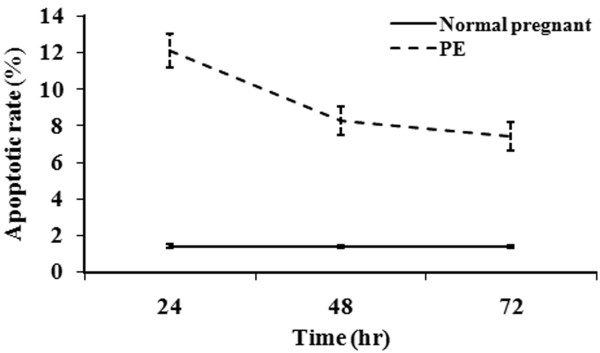
**CRL1730 cell apoptosis after culture in the presence of normal pregnant or PE sera.** Continuous variables were presented as mean ± SD and comparison of effect between two groups over time was performed by repeated measures of ANOVA and difference in apoptotic rate between two groups over time was significant (P < 0.001). n = 20 for each group.

To determine if the decreased cell growth induced by PE serum was due to apoptosis, Annexin-V staining was undertaken (Figures 3 and 4). Representative results for each group and time point assessed are found in Figure 3. Compared with the cells treated with control maternal sera, significantly increased apoptosis was observed in those treated with PE sera at each time point analyzed (*P* <0.01), indicating that PE serum promoted apoptosis (Figure 4). However, the apoptosis rates in the PE group significantly decreased with time (*P* <0.05) while no difference was found in the control group.

### Effects of normal pregnant and PE sera on CD40/CD40L protein and mRNA expression in CRL1730 cells

**Figure 5:**
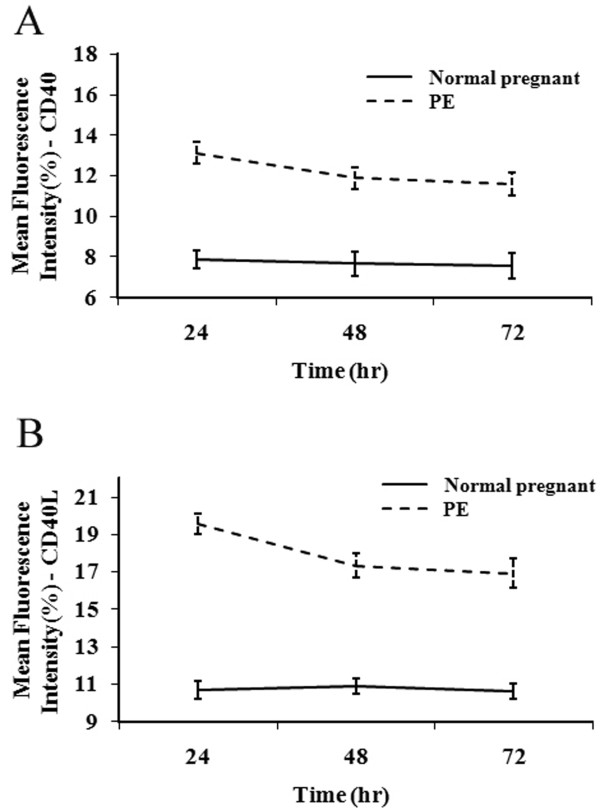
**Effects of normal pregnant and PE sera on CD40/CD40L protein expression in CRL1730 cells.** CD40 (A) and CD40L (B) protein expression in CRL1730 cells after treatment with normal pregnant and PE sera. Continuous variables were presented as mean ± SD and comparison of effect between two groups over time was performed by repeated measures of ANOVA and difference in CD40 and CD40L protein expression in CRL1730 cells between two groups over time was significant (P < 0.001). n = 20 for each group.

The effects of maternal sera from healthy pregnant women and PE patients on CD40 and CD40L protein and mRNA expression were next determined using flow cytometry and RT-PCR analyses, respectively. The expression of both CD40 (Figure 5A) and CD40L (Figure 5B) was observed in each group at all the time points analyzed; however, differences in the extent of expression were noted (Figure 5). Compared with the control group, CD40/CD40L expression increased significantly in the PE group (*P* <0.01). No statistical difference in the expression of CD40/CD40L was found in the control over time (*P* >0.05); however, expression of CD40/CD40L at 24 h was higher than observed at 48 and 72 h in the PE group (*P* <0.05).

**Figure 6:**
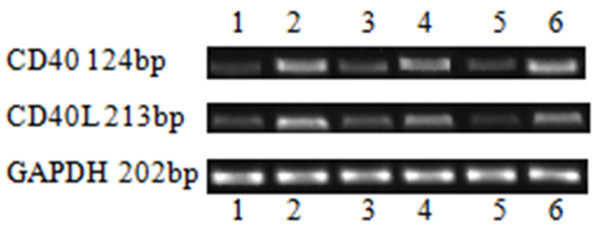
**Upregulation of CD40/CD40L expression upon stimulation with PE sera.** CD40/CD40L mRNA expression was determined in CRL1730 cells treated with serum from normal and PE pregnant women using RT-PCR. Lane 1) 24-h normal pregnant group; lane 2) 24-h PE group; lane 3) 48-h normal pregnant group; lane 4) 48-h PE group; lane 5) 72-h normal pregnant group; and lane 6) 72-h PE group. A representative image from three independent experiments is shown.

**Figure 7:**
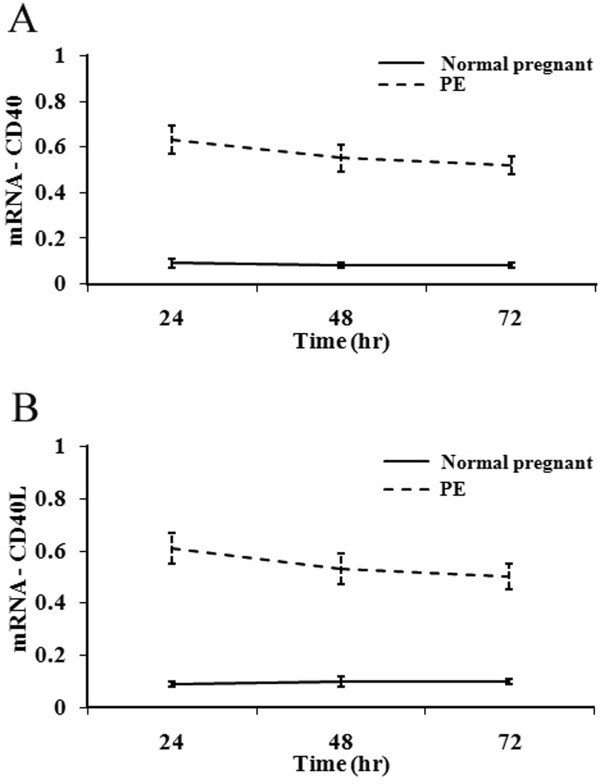
**Effects of normal pregnant and PE sera on CD40/CD40L mRNA expression in CRL1730 cells.** Expression of CD40 (A) and CD40L (B) mRNA in CRL1730 cells cultured in the presence of sera from normal pregnant patients or those with PE. Results were presented as mean ± SD and comparison of effect between two groups over time was performed by repeated measures of ANOVA and difference in expression of CD40 and CD40L mRNA in CRL1730 cells cultured between two groups over time was significant (P < 0.001). n = 20 for each group.

RT-PCR analysis was next undertaken to confirm the upregulated expression of CD40 and CD40L mRNA upon stimulation with PE maternal sera (Figures 6 and 7). CD40 and CD40L mRNA expression was significantly greater in the PE group than in the control group. CD40/CD40L expression was significantly higher at 24 h than at 48 and 72 h (*P* <0.01), and CD40 expression was significantly decreased at 72 h as compared to 48 h (*P* <0.05; Figure 7 A&B). These results indicated that the upregulation of CD40/CD40L expression by maternal sera isolated from PE patients occurs at an early stage.

## Discussion

Studies suggest that endothelial cell dysfunction is the key pathophysiology underlying PE [15]; it may even be an example of an endothelial damage disease [4]. Oxidative stress is a common inducer of endothelial cell damage, resulting in altered function [16]. Inflammatory mediators (e.g., tumor necrosis factors, interleukin-6, very low density lipoproteins, etc.) may trigger oxidative stress and result in endothelial damage.

Various pathogenic factors can cause placental ischemia and hypoxia, triggering the release of vasoactive substances. Systemic small vessel spasm and injury ensues, damaging vascular endothelial cells. Placental oxidative stress in PE is also associated with increased anti-angiogenic factors, including soluble fms-related tyrosine kinase-1 (sFlt-1) and soluble endoglin, which may be due to overexpression of growth arrest and DNA damage-inducible 45 alpha (Gadd45α) and excessive activation of p38 mitogen-activated protein kinase (MAPK); knockdown of their expression resulted in decreased endothelial cell apoptosis and oxidative stress and increased in vitro angiogenesis [17]. Further studies will assess the role of Gadd45α and p38 in endothelial cell functional changes observed upon stimulation with PE sera.

Increased serum markers of endothelial cell dysfunction, including E-selectin and von Willebrand factor, have been observed in patients with PE [18]. In the present study, altered CRL1730 cell morphology, growth and apoptosis were observed upon treatment with PE sera, which is indicative of endothelial cell dysfunction. However, endothelial cell function was not directly analyzed; therefore, further studies are necessary to determine the full extent of the damage induced by PE sera. Szarka *et al.*[19] showed that the levels of pro-inflammatory cytokines, chemokines, and adhesion molecules were elevated in the maternal serum of PE patients compared to healthy pregnant women. The excessive systemic inflammatory response might be one of the factors leading to the endothelial dysfunction/activation.

Increased serum heat shock protein 70 (Hsp70) levels have been observed in patients with early-onset or severe PE [20]. Hsp70 along with other pro-apoptotic proteins have been shown to mediate endothelial cell apoptosis in PE subjects [21], which is in agreement with the increased CRL1730 cell apoptosis upon culture with maternal sera obtained from PE patients. However, the apoptosis rate decreased over time. Although a higher concentration of soluble CD40L was shown to protect maternal immune cells bearing CD40 receptor from Fas-mediated apoptosis [22], the decreased apoptosis rate could be not explained by decreased CD40/CD40L in the present study. Degradation and/or catabolism of Hsp70 and other pro-apoptotic molecules present in the PE serum may account for this observation.

In the present study, increased expression of CD40/CD40L was observed in the early stage of stimulation with PE sera, suggesting the involvement of CD40/CD40L in the occurrence of early-onset PE. This observation is consistent with the involvement of CD40/CD40L in early coronary atherosclerosis [23]. It is also consistent with the study of Darmochwal-Kolarz *et al.*[24] in which the serum concentration of soluble CD40L was significantly higher in PE women as compared to healthy pregnant women. Its reduced expression in healthy pregnant women as compared to non-pregnant women is suggestive of a decreased innate immunity in normal pregnancy that is disturbed in PE [24].

The present study has limitations. Firstly, cell growth was measured using an MTT assay, which provides a measure of mitochondrial activity and requires cell digestion; however, the actual cell numbers were not quantified due to limited blood sample volume. Further studies using hemocytometry or flow cytometry will be undertaken to directly evaluate cell growth. Furthermore, although changes in CRL1730 growth, apoptosis, and CD40/CD40L expression were observed upon stimulation with PE sera, the serum factors responsible for those changes were not determined in the present study due to the limited blood sample volume obtained. However, further investigations into the responsible factors will be undertaken in future studies. Finally, the role of CD40/CD40L expression will be fully elucidated using neutralization or knockdown studies.

## Conclusion

In summary, the upregulation of CD40/CD40L expression in endothelial cells treated with maternal sera from PE patients coincides with vascular endothelial cell apoptosis. Further studies are necessary to determine the factor(s) mediating these effects as well as elucidate their role in the pathogenesis of PE.

## Competing interests

The authors declare that they have no competing interests.

## Authors’ contributions

CFW, guarantor of integrity of the entire study, drafted the manuscript. FDH contributed to data acquisition and statistical analysis. RFS participated in experimental studies. JXS contributed to supervision. All authors read and approved the final manuscript.
